# Loss of cell polarity regulated by PTEN/Cdc42 enrolled in the process of Hepatopulmonary Syndrome

**DOI:** 10.1111/jcmm.14437

**Published:** 2019-05-29

**Authors:** Jing Gao, Hongfu Yu, Xuehong Bai, Chang Liu, Lin Chen, Karine Belguise, Xiaobo Wang, Kaizhi Lu, Zhiyong Hu, Bin Yi

**Affiliations:** ^1^ Department of Anaesthesia Southwest Hospital, The Third Military Medical University (Army Medical University) Chongqing China; ^2^ Department of Anaesthesia The Children’s Hospital, Zhejiang University School of Medicine Hangzhou China; ^3^ LBCMCP, Centre de Biologie Intégrative (CBI) Université de Toulouse, CNRS, UPS Toulouse France

**Keywords:** Cdc42, cell polarity, Hepatopulmonary Syndrome, PMVEC, PTEN

## Abstract

One central factor in hepatopulmonary syndrome (HPS) pathogenesis is pulmonary vascular remodelling (PVR) which involves dysregulation of proliferation and migration in pulmonary microvascular endothelial cells (PMVECs). Growing evidence suggests that Apical/basolateral polarity plays an important role in cell proliferation, migration, adhesion and differentiation. In this study, we explored whether cell polarity is involved and critical in experimental HPS rats that are induced by common bile duct ligation (CBDL). Cell polarity related proteins were analysed in CBDL rats lung and PMVECs under the HPS serum stimulation by immunofluorescence assay. Cdc42/PTEN activity, cell proliferation and migration and Annexin A2 (AX2) in PMVECs were determined, respectively. Cell polarity related proteins, lost their specialized luminal localization in PMVECs of the CBDL rat. The loss of cell polarity was induced by abnormal activity of Cdc42, which was strongly enhanced by the interaction between *p*‐PTEN and Annexin A2 in PMVECs, after treatment with serum from CBDL rats. It led to over‐proliferation and high migration ability of PMVECs. Down‐regulation of PTEN‐Cdc42 activity in PMVECs restored cell polarity and thus reduced their ability of migration and proliferation. Our study suggested that the loss of cell polarity plays a critical role in the pathogenesis of HPS‐associated PVR and may become a potentially effective therapeutic target.

## 1 INTRODUCTION

There are 100 million hepatitis B virus carriers in China. Approximately 20%–40% of them developed hepatitis‐cirrhosis and 20%–30% of these hepatitis‐cirrhosis patients end up with Hepatopulmonary Syndrome (HPS)^1^, ^2^ which significantly increases mortality. At present, the pathophysiological mechanism underlying is unclear and liver transplantation is the only treatment option. Pulmonary vascular remodelling (PVR), which impairs arterial oxygenation in the setting of chronic liver diseases, is the key pathophysiological component of HPS.^3^ In our previous studies, we established the role of cell proliferation, migration and phenotype differentiation in PVR associated with HPS.^4^, ^5^, ^6^, ^7^

Cell apical/basolateral polarity is of fundamental importance for the processes of cell division,^8^ differentiation,^9^ proliferation^10^, ^11^ and migration^12^, ^13^, ^14^ in multicellular organisms. The circulating cytokines that are released by diseased liver result in the activation of multiple signalling pathways, such as PI3K, PKGIa, JAK/STAT and PKC/ERK, which directly or indirectly participate in regulating cell polarity. AmotL2 increase,^13^ β‐catenin depletion,^15^ PI3K and SHIP2 deregulation^16^ all attenuate cell‐cell adherins junction of pulmonary microvascular endothelial cells (PMVECs) and induce the loss of cell polarity, which ultimately results in abnormal cell proliferation and migration. The signalling pathway involved in may be pivotal for HPS‐associated PVR, and blocking the pathway may effectively reduce the incidence and development of PVR. Thus, it is important to characterize specific molecules that might control the multiple cellular activities required for cell polarization and to elucidate their regulatory mechanism in the process of PVR in HPS.

Many previous studies supported the critical role of Cdc42 in control of apical‐basolateral polarity in different systems.^17^, ^18^ In this study, we demonstrated that the loss of apical/basolateral polarity in PMVECs occurred in the process of PVR associated with HPS. This cell polarity loss was mediated by the enhanced PTEN‐Annexin A2(AX2)‐Cdc42 signalling pathway, and the down‐regulation of this signalling pathway can restore cell polarity and reduce the abnormal migration and proliferation ability of PMVECs in response to common bile duct ligation (CDBL) rat serum.

## 2 MATERIALS AND METHODS

### 2.1 Reagents

Antibodies against β‐catenin, podocalyxin (PCX) and AX2 as well as the Cdc42/RhoA/Rac1 activation assay and PTEN inhibitor BpV(pic) were purchased from Abcam (Cambridge, UK). The antibody against gp200 and the Pierce™ Co‐Immunoprecipitation Kit was obtained from Thermo Fisher Scientific (Waltham, US). The antibodies against PTEN and Alexa Fluor® 488 Phalloidin as well as cell lysis buffer were obtained from Cell Signalling Technology (Beverly, MA). Dulbecco's modified Eagle's medium (DMEM, high glucose) and Foetal bovine serum (FBS) were purchased from Gibco (Waltham, MA, US). NuPAGE Novex 4%–12% Bis–Tris Gels and Lipofectamine 2000 were purchased from Invitrogen (Carlsbad, CA). Transwell chambers were purchased from Corning, Inc (New York, US). The Cdc42 inhibitor, Casin and the β‐actin antibody were purchased from Sigma‐Aldrich (San Francisco, US).

### 2.2 Animals

Male Sprague‐Dawley (SD) rats (200‐250 g, Third Military Medical University, Chongqing, China) were used in all of the experiments. All procedures performed on the rats were conducted according to guidelines from the National Institutes of Health. The study protocol was approved by the committee on Animal Research of Southwest Hospital. CBDL was performed to establish an experimental HPS rat model as previously described. Four weeks after surgery, the rats were used as the standard model of HPS.^19^ The sham animals underwent common bile duct exposure but no ligation. Specimens were collected at the end of 1, 2, 3 and 4 weeks. CBDL rat serum was collected from rats that had developed HPS. Normal rat serum was collected from the sham rats at the same time. All of the serum samples were used for subsequent experiments.

### 2.3 Cell culture and transfection

PMVECs were isolated from normal SD rat lungs as previously described.^20^ Experimental data were obtained from cells between passages two to six. PMVECs were divided into two groups as follows: the sham group consisted of PMVECs that were cultured in DMEM supplemented with normal rat serum (5%); and the CBDL group consisted of PMVECs that were incubated in DMEM containing 5% CBDL rat serum (1, 2, 3 and 4 weeks) for 24 hours.

For drug treatment groups, PMVECs were added with 0.05 μM PTEN inhibitor BpV(pic) (Abcam，Cambridge, UK) or 5 μM Casin (Sigma‐Aldrich) for 24 hours in different groups. PMVECs were transfected at 70% confluence for 36 hours with 50 nM short interfering (si)RNAs targeting rat AX2 or a negative control siRNA (NC siRNA) using HiPerfect reagent (Qiagen). Briefly, the siRNA was complexed with 20 μL of transfection reagent and diluted with M199 to 120 μL. Complete fresh medium was added at 3 hours after transfection, and the cells were further incubated for 24‐36 hours before administration of subsequent treatments.

### 2.4 Transepithelial electrical resistance (TER) assay

Transepithelial electrical resistance measurements of PMVEC monolayers grown in Transwell filter units (0.4 μm pore size and 6.5 mm diameter) were utilized according to a protocol described previously.^21^ Transepithelial resistance (ohm‐square centimetres) was calculated by multiplying the measured electrical resistance by the area of the filter (0.33 cm^2^), and TER measurements were recorded prior to changes of media at 3, 6, 9, 12, 24, 30 and 36 hours after the addition of treatments. Treatments were performed in duplicate and repeated three times for the entire experiment. Values were expressed as ohms per square centimetre (Ω/cm^2^).

### 2.5 Western blot analysis

Total proteins of PMVECs and lung tissue were extracted using a protocol described previously.^7^ Equal amounts of proteins (40 μg) were separated through 6%–10% SDS‐PAGE and subsequently electroblotted onto polyvinylidene fluoride membranes (Bio‐Rad Laboratories). After blocking with 5% skim milk in Tris‐buffered saline/Tween buffer (TBST buffer) for 1 hour, the membranes were incubated with anti‐rabbit antibodies against *p*‐PTEN (dilution 1:1,000; CST), PTEN (dilution 1:1,000;CST), and PI3K (1:1,000 dilution; CST). The membranes were incubated with horseradish peroxidase‐conjugated secondary antibodies and visualized using an ECL detection kit (Applygen, Beijing, China) according to the manufacturer's instructions. The same membrane was stripped and reblotted with anti‐mouse or ‐rabbit antibodies against β‐actin (1:10,000 dilution; Sigma‐Aldrich). Finally, the membranes were visualized using a gel imaging system (Bio‐Rad Laboratories, Hercules, CA, USA). The optical density of immunoreactivity was analysed with an Alpha Imager (Protein Simple, San Francisco, CA, USA).

### 2.6 Immunofluorescence microscopy

Immunofluorescence was performed as described previously using FITC‐, or Alex 488‐conjugated secondary antibodies (Abcam).^22^ Confocal microscopy was performed at room temperature (RT) on a microscope (model LSM 510; Carl Zeiss MicroImaging, Inc) and a TCS SP2 system (Leica; 63 oil immersion objectives, NA 1.4). Immunofluorescence images were taken sequentially, and parameters were adjusted so that all light intensities were in the recording range. Confocal stacks were acquired with a slice thickness of 300 nm. Micrographs were derived as indicated in the figure legends from single confocal planes, several merged confocal planes, vertical sections generated from confocal stacks with the LSM software (Carl Zeiss MicroImaging, Inc), or as a merged image of several vertical sections generated by reslicing of a confocal stack with Image J software (W. Rasband, National Institutes of Health, Bethesda, MD). Pictures were arranged with Adobe Photoshop and Adobe Illustrator. Experiments were performed at least in triplicate, and representative images of highly expressing cells are shown. Similar experiments were performed using PMVECs transfected with siRNAs or treated with inhibitor.

### 2.7 Rho GTPase pull down assays

The preparation of GST fusion proteins and the pull down assay for active Rac1, Cdc42 and RhoA was performed according to a previously reported procedure.^23^ Briefly, PMVECs were grown in six wells culture‐plates for 2 days and stimulated with 5% normal or CBDL rat serum (4 weeks). For treatment groups, PMVECs were added with 0.05 μM PTEN inhibitor BpV(pic) (Abcam) or 5 μM Casin (Sigma‐Aldrich) for 24 hours in different groups. After treatment, the cells were washed with ice‐cold PBS and scraped into 0.3 mL of lysis buffer. The lysates were cleared by centrifugation. A sample from the supernatant was set aside for determination of total level of GTPases, and equal volumes of lysates were then incubated with Rhotekin‐RBD –agarose (for RhoA‐GTP) or PAK‐1 PBD–agarose (for Cdc42 or Rac1‐GTP) for 1 hour at 4°C followed by three washes with lysis buffer. The beads were boiled in sodium dodecyl sulphate (SDS) sample buffer. Total amounts of Rho proteins from cell lysates were separated by SDS–polyacrylamide gel electrophoresis (PAGE) and measured by Western blotting using specific antibodies. The amount of the GTP‐bound form was normalized to the total amount of Rho GTPase in cell lysates. All experiments were performed three times independently.

### 2.8 Immunoprecipitation

Protein expression was assessed through Western blotting. Briefly, PMVECs were treated with 5% normal rat serum or 5% HPS rat serum for 24 hours. Cells were lysed in modified radioimmunoprecipitation assay (RIPA) buffer (50 mM Tris, pH 7.2; 150 mM NaCl, 0.25% deoxycholate; 1% NP‐40; and protease and phosphatase inhibitors). Cell extracts were precleared with 50 μL of protein G Plus/A agarose beads (Calbiochem, San Diego, CA) for 30 minutes at 4°C. Protein concentration was determined using the BCA Protein Assay Reagent Kit (Bio‐Rad Laboratories, Hercules, CA) according to the manufacturer's instructions. Two micrograms of antibody was incubated with 500 μg of extract for 2 hours at 4°C with gentle mixing. Fifty microlitres of protein G Plus/A agarose beads was added, and the immune complexes were incubated for 1 hour at 4°C. Immunoprecipitates were washed three times with 0.5 mL of modified RIPA buffer, mixed with sample buffer and separated by SDS–PAGE for immunoblot analysis. Loading was confirmed by stripping and reprobing the blot with GAPDH. The bands were visualized with chemiluminescence detection (ECL, GE Health Care, Westborough, MA). Quantitative analysis was performed using ImageJ Software. All experiments were performed three times independently.

### 2.9 Cell migration assay

Cell migration was examined using a Transwell chamber assay with 24‐well chambers (pore size 8 μm; Corning). For drug treatment groups, PMVECs were added with 5 μM Casin (Sigma‐Aldrich) for 24 hours in different groups. Approximately 500 μL of DMEM medium containing 5% serum was added to the lower wells, and PMVECs were seeded at 10 × 10^4 ^cells onto the upper wells containing 300 μL of DMEM medium without serum. The chambers were subsequently incubated for 12 hours and 24 hours at 37°C. The cells were fixed in 4% polyoxymethylene solution for 5 minutes and stained with 1% crystal violet. The migrated cells were manually calculated in three random microscopic fields using a fluorescence microscope.

### 2.10 Cell proliferation assays

Cell proliferation was measured and quantified by Ki‐67 staining following each treatment. PMVECs were added with 5 μM Casin (Sigma‐Aldrich) for 24 hours in different groups.Six‐μm‐thick paraffin sections of fixed PMVECs were blocked by 10% bovine serum albumin for 2 hours. Next, the sections were incubated overnight at 4°C with primary antibody, Ki‐67 (1:1000，abcam, Cambridge, MA, ab15580).For immunofluorescence staining of Ki‐67, fluorescence‐tagged secondary antibody and 4′, 6‐diamidino‐2‐phenylindole were used. The section was investigated using a confocal microscope. Each assay was performed three times in triplicate.

### 2.11 Statistical analysis

Measurements are expressed as arithmetic means ± SD. Data were normally distributed with homogeneous variances. Therefore, multiple group comparison testing was performed using SPSS 17.0 software and determined using one‐way ANOVA followed by the Student‐Newman‐Keuls post‐hoc test. A value of *P* < 0.05 was considered statistically significant.

## 3 RESULTS

### 3.1 The loss of apical/basolateral polarity occurs in the CBDL rat lung tissue in vivo and the cultured PMVECs in vitro

**Figure 1:**
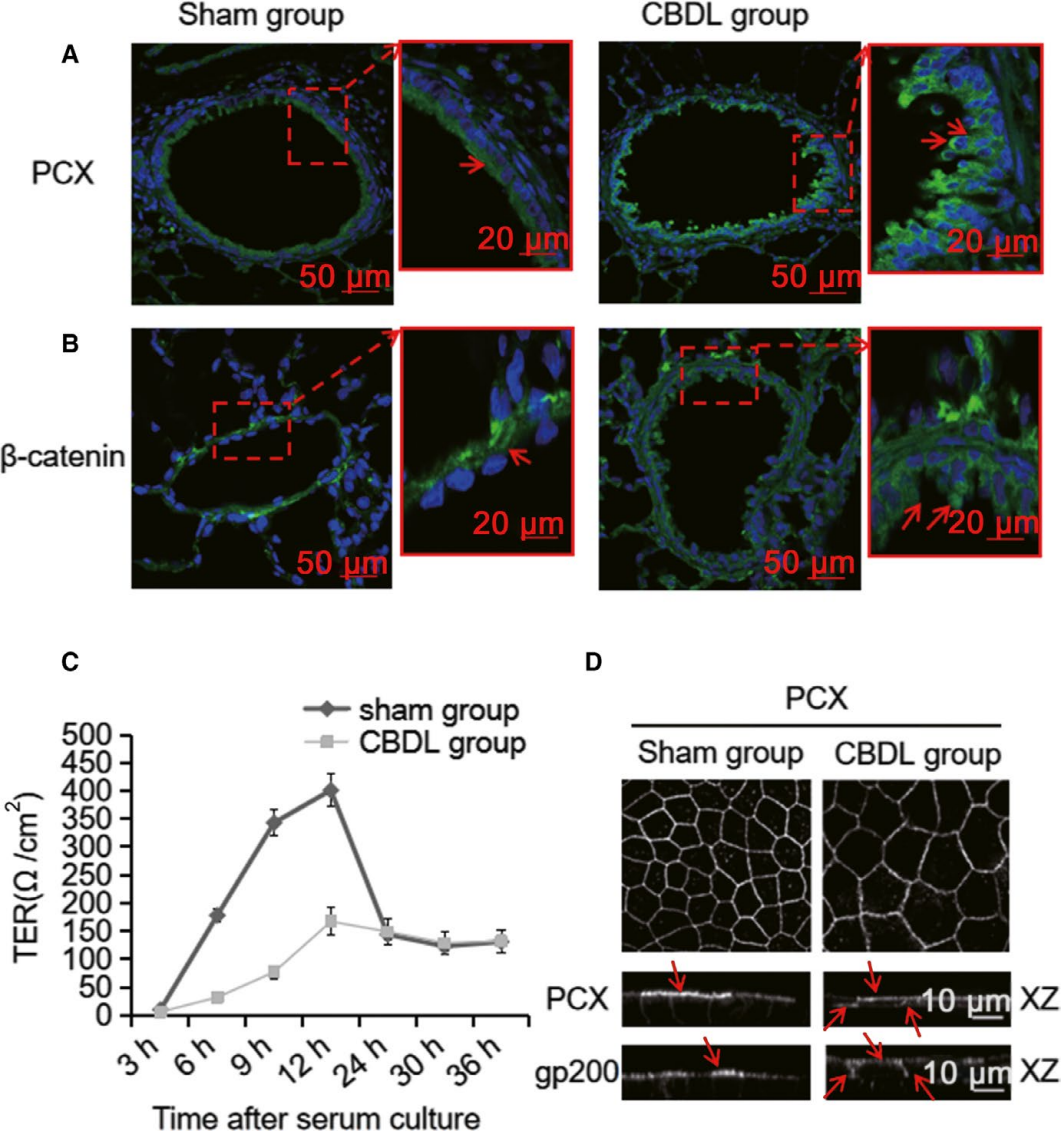
The mis‐location of cell polarity proteins exists in PMVECs of CBDL rat lung tissue or cultured PMVECs. A, Immunofluorescence labelling was performed to evaluate the distribution of cell polarity proteins. PCX was broadly redistributed and presented around the entire cell perimeter in the CBDL group, while the labelling of PCX was only distributed in the apical membrane in the sham group (arrows). B, β‐catenin, which was restricted to the basolateral surface of vessels in the sham group (arrows), lost its specialized localization in the CBDL group. C, Transepithelial resistance readings (Ω/cm^2^) of PMVECs at 3, 6, 9, 12, 24, 30 and 36 h after the addition of treatments. Cell‐cell tight junction formation was reduced by CBDL rat serum compared to the sham group at the same time, especially at 9 h. D, Immunofluorescence analysis of cultured PMVECs. Cells in the sham and CBDL groups were seeded onto filters and fixed 2 d after reaching confluency. A confocal midsection stained for the apical marker, PCX, showsed that cells of the CBDL group were localized on the cell surface. The corresponding vertical section showed that cells of the CBDL group did not restrict PCX to the apical surface similar to the cells of the sham group. The apical marker protein, gp200, also displayed a less polarized distribution in the CBDL group

In our CBDL rat models, we observed abnormal localization of cell polarity proteins in PMVECs of CBDL rat lung tissue by the histological examination of the polarity protein distribution. Podocalyxin (PCX), an apical marker of endothelium, is restricted to the apical surface of vessels.^22^ Consistently, we observed the apical distribution of PCX around vessels in the control sham rat lung tissue while it was broadly redistributed and present around the entire endothelial cell perimeter in the CBDL rat lung tissue (Figure 1A, arrows). Another polarized cell‐cell adhesion protein, β‐catenin, was localized to the endothelial cell‐cell adhesion regions of vessels in the control sham rat lung tissue but lost its specialized endothelial localization in the CBDL rat lung tissue (Figure 1B, arrows). Moreover, endothelial cells in the inner surface of CBDL rat lung vessels displayed irregular arrangement and were multi‐layered (Figure 1A and B) and the number of vascular endothelial cells was significantly increased in the CBDL rat group(Figure 1A and B).

Next, we checked whether the loss of endothelial cell polarity can also be observed in the cultured PMVECs, under the stimulation with CDBL rat serum (from 4 weeks CBDL rat). Thus, in cultured PMVECs, we determined the distribution of endogenous apical polarity marker proteins such as PCX and glycoprotein CD200(gp200). They were both correctly distributed at the apical domain of PMVECs under the stimulation with normal rat serum (Figure 1D, left panel arrows). Surprisingly, PCX and gp200 were detected at both the apical and basolateral domains of PMVECs, under the stimulation with CBDL rat serum (Figure 1D, right panel arrows). These indicated that the loss of apical/basolateral polarity occurred both in vivo and in vitro.

Since cell‐cell tight junctions are the foremost fundamental structures of cell polarity and indirectly reflect cell polarity formation, we further characterized it by Transepithelial electrical resistance (TER) assay. Compared with the sham group, the presence of CBDL rat serum reduced the cell‐cell tight junction formations in PMVECs, especially at 9 hours, while no significant difference was observed after 24 hours (Figure 1C).

### 3.2 CBDL rat serum induces the disruption of apical/basolateral polarity via the activation of Cdc42

**Figure 2:**
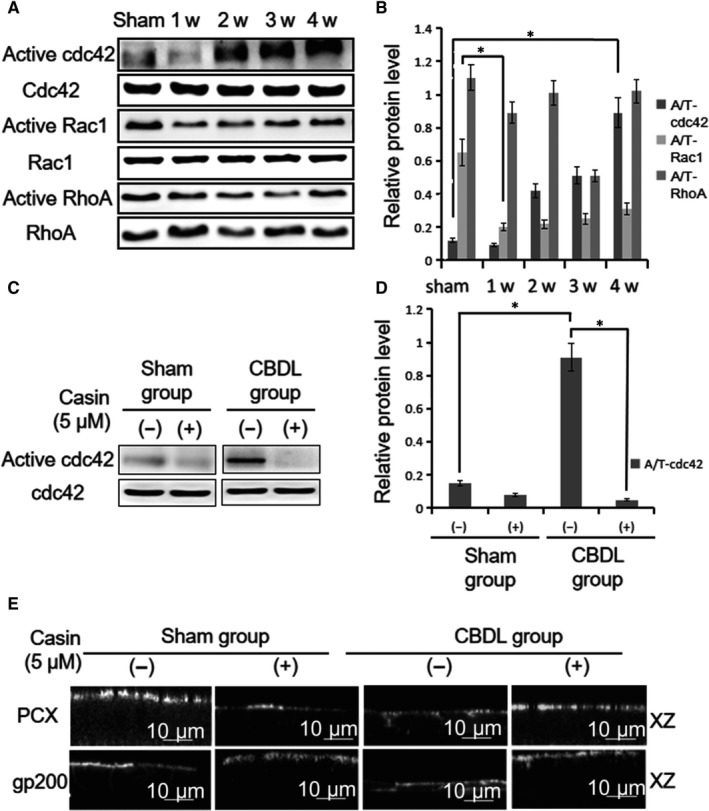
CBDL rat serum induces cell polarity defects via Cdc42*.* A, The pull down assay showed that CBDL rat serum influenced the activity of Cdc42 in vivo. The activity of Rac1 was reduced in the CBDL group. However, the activity of RhoA in the CBDL group maintained a similar level to that in the sham group. B, The level of relative protein activity normalized to total protein is displayed by bar charts. C, Cultured PMVECs were pre‐treated with Casin (a selective inhibitor of Cdc42) or vehicle solutions. The result of immunoblotting showed that treatment with CBDL rat serum increased the activity of Cdc42. However, the activity of Cdc42 was inhibited in the presence of Casin. D, The level of active Cdc42 relative to total Cdc42 is displayed. E, Localization of PCX and gp200 in PMVECs was captured by immunofluorescence microscopy. Casin‐mediated inhibition of Cdc42 overactivation in PMVECs recovered the distribution of PCX and gp200 from the abnormal area to the correct apical domain. (−):vehicle solutions;(+):Casin treatment;*P < 0.05

Our next question is how the loss of endothelial apical/basolateral polarity occurs. Rho small GTPases have been known to play a central role in establishing apical/basolateral polarity in endothelial cells and epithelial cells. To investigate whether the activity of Rho small GTPase family proteins shows any change during PVR associated with HPS, cell lysates of lung tissue from CBDL rat and sham rat were assayed for the levels of active RhoA, Rac1 and Cdc42. The levels of active Cdc42 were increased and the levels of active Rac1 were reduced in CBDL group(*P* < 0.05) (Figure 2A and B). However, RhoA activities in CBDL rat lung tissue maintained a similar level to that in the sham group (*P* > 0.05) (Figure 2A and B). Consistent with our in vivo observation, the activity of Cdc42 in PMVECs was remarkably increased after the stimulation with CBDL rat serum(*P* < 0.05) (Figure 2C and D). Both in vivo and in vitro results indicated that the enhanced Cdc42 activity might lead to the loss of endothelial apical/basolateral polarity.

To investigate whether overactivation of Cdc42 regulates the disruption of apical/basolateral polarity in PMVECs treated with CBDL rat serum, PMVECs were pre‐treated with Casin, a selective inhibitor of Cdc42 activity.^24^ Indeed, pre‐treatment of Casin strongly blocked the overactivation of Cdc42 in PMVECs (*P* < 0.05) (Figure 2C and D). Importantly, the Casin‐mediated inhibition of Cdc42 overactivation in PMVECs recovered the distribution of PCX and gp200 from the abnormal area to the correct apical domain (Figure 2E). These results indicated that the disruption of apical/basolateral polarity were induced by CBDL rat serum via the overactivation of Cdc42.

### 3.3 The recovery of apical/basolateral polarity by Cdc42 inhibition is accompanied with the changes in actin cytoskeleton reassembly, cell proliferation and cell migration

**Figure 3:**
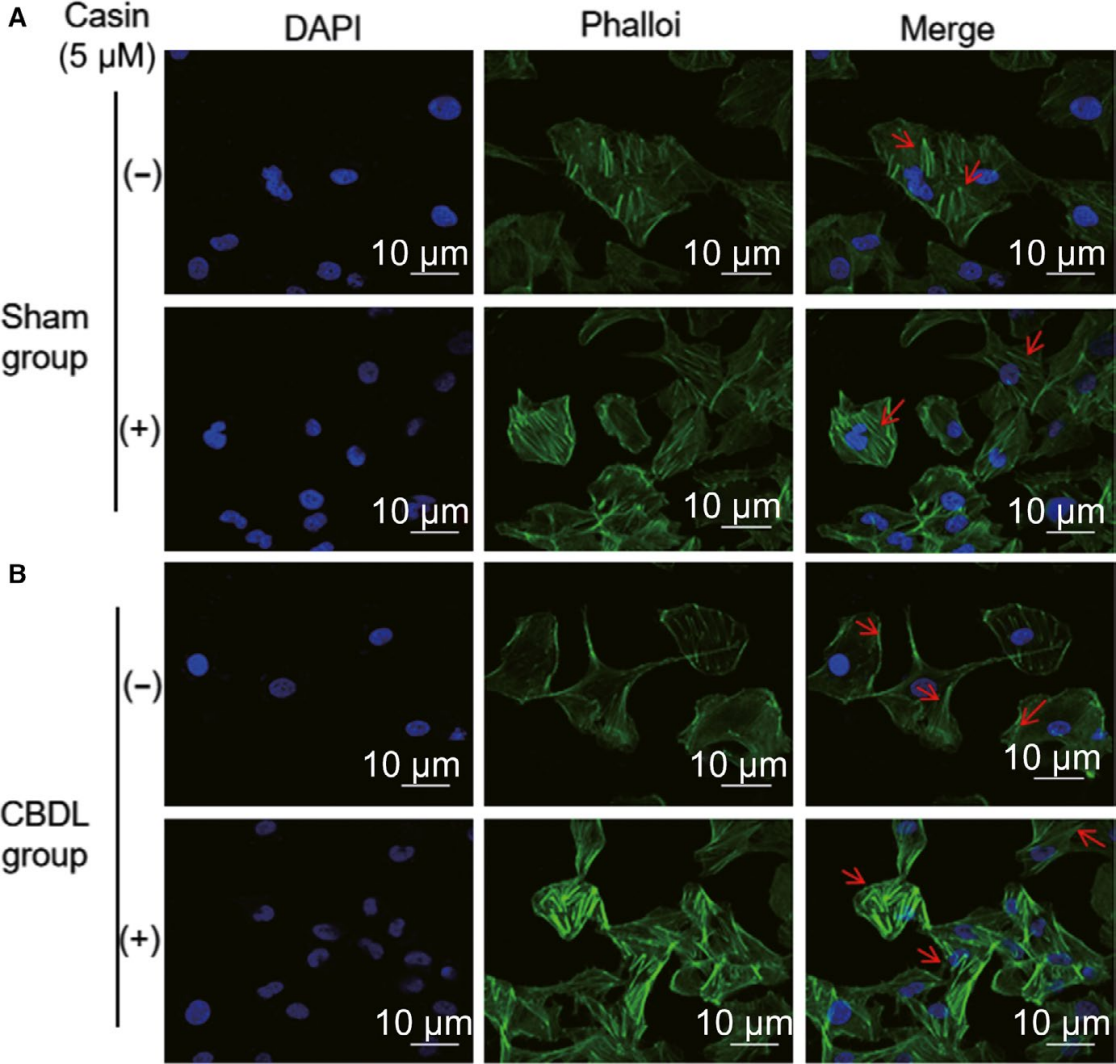
Cdc42 inhibitor reverses cell polarity defects and is accompanied by changes in actin cytoskeleton reassembly in vitro. F‐actin was stained with FITC‐conjugated phalloidin in each group. Images were taken by confocal microscopy. A, Basic lamellipodia protrusions around the circumference of cultured PMVECs in sham group, with intact actin stress fibres (arrows). B, PMVECs cultured in the presence of CBDL rat serum displayed an unstable phenotype with the large filopodia protrusions outside of extended lamellipodia membrane. Filopodia protrusions were completely lost and lamellopodia membrane was recovered back to basic levels, and stress fibers (arrows) were strongly enhanced after Casin treatment. (−):vehicle solutions;(+):Casin treatment;DAPI:4′, 6‐diamidino‐2‐phenylindole

Cell apical/basolateral polarity, migration and proliferation are all dependent on the rearrangement of the actin cytoskeleton.^23^ Thus, we assessed the actin cytoskeleton by visualizing it with the FITC‐conjugated phalloidin, and investigated the effect of CBDL rat serum on the actin cytoskeleton in PMVECs. F‐actin staining showed that PMVECs stimulated with sham rat serum had some basic lamellipodia protrusions around their circumference, and also exhibited intact actin stress fibres (Figure 3A). In contrast, PMVECs stimulated with CBDL rat serum displayed an unstable phenotype with the large filopodia protrusions outside of extended lamellipodia membrane, typical of unpolarized and migrating endothelial cells, while stress fibres were in basic low levels (Figure 3B). After Casin (Cdc42 inhibitor) treatment, filopodia protrusions in PMVECs were completely lost and lamellopodia membrane were recovered back to basic levels, and stress fibres were strongly enhanced (Figure 3A and B). These results indicated that the Cdc42 inhibition, possibly via the control of endothelial cell apical/basolateral polarity, recovered the non‐migrating phenotype mediated by the rearrangement of the actin cytoskeleton networks in PMVECs.

**Figure 4:**
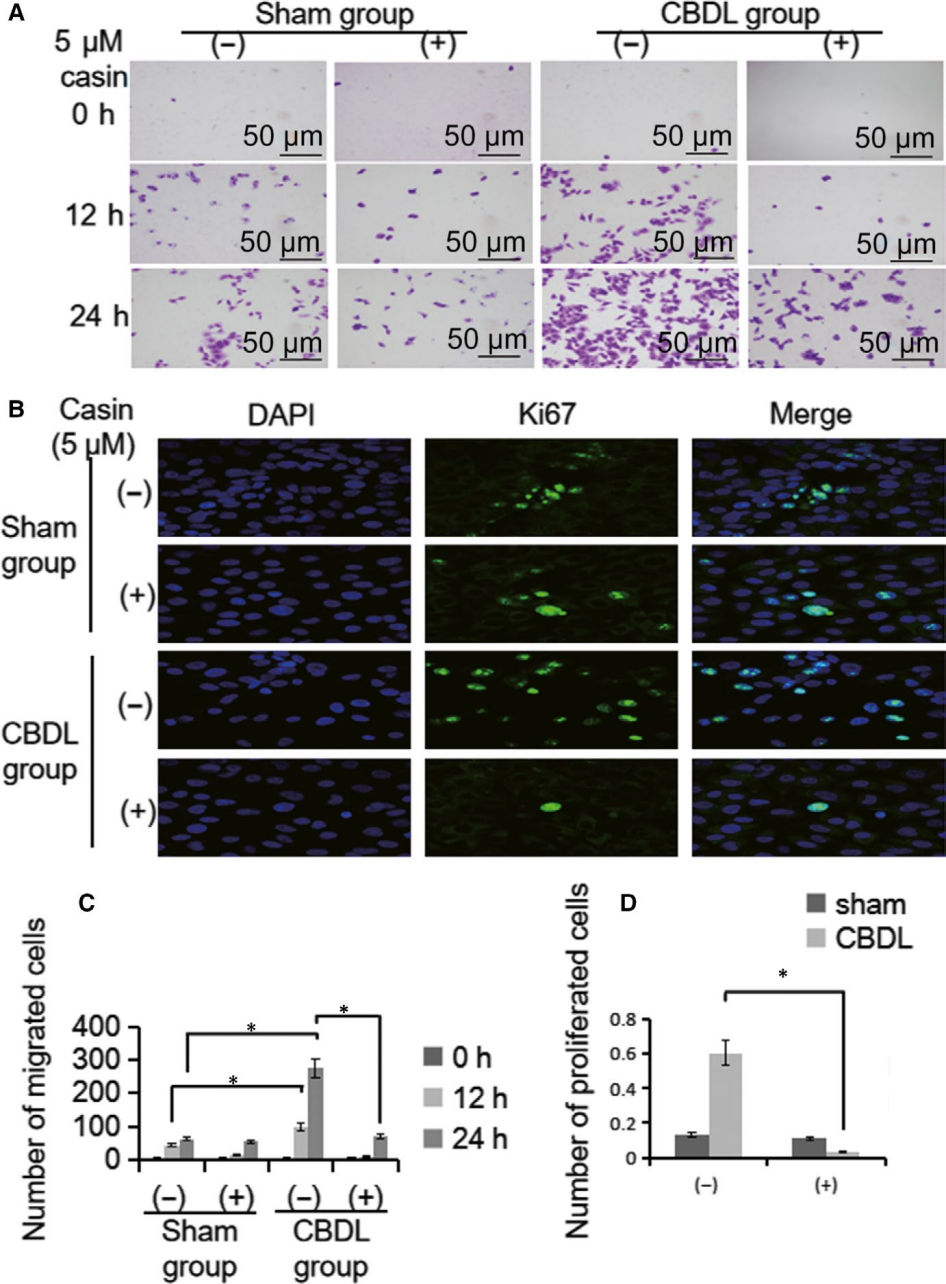
Cdc42 inhibitor reverses cell polarity defects and is accompanied by changes in cell migration and cell proliferation in vitro. A, Transwell chamber assay for cell migration. CBDL rat serum effectively induced PMVEC migration. The number of migrated cells was decreased in response to Casin in the CBDL(+) groups compared to those in the CBDL(−) groups. B, Ki‐67 staining for cell proliferation assay. CBDL rat serum effectively promoted PMVEC proliferation. The number of proliferating cells was decreased in response to Casin compared to those in the CBDL(−) groups. C and D, The number of migrating and proliferating cells was measured. Data are represented as the mean ± SD values for at least three independent experiments. (DAPI:4′, 6‐diamidino‐2‐phenylindole (−):vehicle solutions;(+):Casin treatment;*CBDL rat serum significantly increased PMVEC migration and proliferation compared to the sham group; CBDL rat serum stimulated PMVEC migration in a time‐dependent manner; *P* < 0.05)

As HPS‐associated PVR is mainly due to cell overproliferation and high migration ability, which has been often linked with the disruption of cell apical/basolateral polarity.^11^, ^14^ We wondered whether the rearrangement of the actin cytoskeleton and the apical/basolateral polarity loss by overactive Cdc42 were related to the changes of proliferation and migration abilities of PMVECs. We inhibited Cdc42 activity, by the treatment of Casin with the stimulation of CBDL or sham rat serum in PMVECs. The number of migratory PMVECs was increased significantly in response to CBDL rat serum, in a time‐dependent manner (0, 12 and 24 hours) (*P* < 0.05) (Figure 4A and C). Similarly, the number of proliferating PMVECs cells was also increased markedly after CBDL rat serum treatment(24 hours)(*P* < 0.05) (Figure 4B and D). Consistent with our hypothesis, the migratory and proliferating ability were both significantly attenuated after Cdc42 inhibitor treatment (*P* < 0.05) (Figure 4). Taken together, it indicated that the abnormal cell migration and proliferation of PMVECs, was mediated by the overactivation of Cdc42.

### 3.4 Activated PTEN regulates the abnormal activation of Cdc42 in response to CBDL rat serum

**Figure 5:**
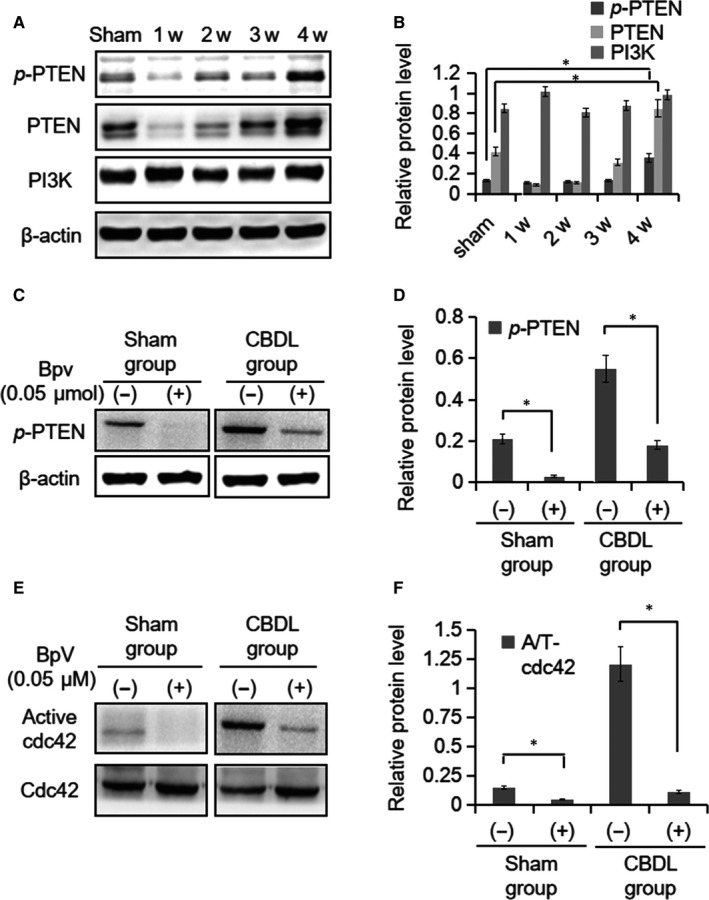
Activated PTEN regulates the activity of Cdc42 in CBDL rat. A, High expression of *p*‐PTEN in lung tissue of CBDL rats at 4 weeks. However, the expression of PI3K was maintained at a similar level to that in the sham group. B, The densitometry of the protein expression compared to that of β‐actin is displayed. 4‐week CBDL rat lung exhibited markedly the *p‐*PTEN expression while PI3K did not show significant changes. C, 0.05 μM PTEN inhibitor BpV(pic) was added in PMVECs for 24 h in different groups and it inhibited the phosphorylation of PTEN. D, The densitometry of the *p‐*PTEN protein expression compared to that of β‐actin is displayed. E and F, The activity of Cdc42 was inhibited in the presence of BpV(pic). (−):vehicle solutions;(+):Bpv(pic) treatment;*P < 0.05

The following question is how Cdc42 is overactivated in PMVECs of CBDL rat lung tissue in vivo, or PMVECs with the stimulation of CBDL rat serum in vitro. Since PTEN has been known to be an important regulator of Cdc42 activity, we determined if PTEN regulates the disruption of cell apical/basolateral polarity in PMVECs both in vivo and in vitro*.* Four‐week CBDL rat lung exhibited markedly the activated PTEN expression (*P* < 0.05). However, the expression of PI3K, another important control of cell proliferation, did not show significant changes in the 4‐week CBDL group (*P* > 0.05) (Figure 5A and B). Consistent with the in vivo obserevation, PMVECs with the stimulation of CBDL rat serum showed significant increase in the activated PTEN levels (Figure 5C and D). Taken together, our data indicated that active PTEN might lead to the overactivation of Cdc42 in CBDL rat lung and PMVECs with the stimulation of CBDL rat serum.

To determine if increased activity of Cdc42 might be controlled by PTEN activation, Cdc42 activity was analysed in PMVECs after treatment with BpV(pic), an inhibitor of PTEN by blocking the phosphorylation of PTEN.^25^ First, we confirmed that BpV(pic) treatment successfully inhibited the phosphorylation of PTEN (*P* < 0.05) in PMVECs (Figure 5C and D). Consistent with our hypothesis, the levels of activated Cdc42 diminished in the CBDL group (*P* < 0.05) (Figure 5E and F). It suggested that CBDL rat serum possibly disrupted the apical/basolateral polarity in PMVECs via activating PTEN‐Cdc42 signalling axis.

### 3.5 PTEN activates Cdc42 in PMVECs via AX2

**Figure 6:**
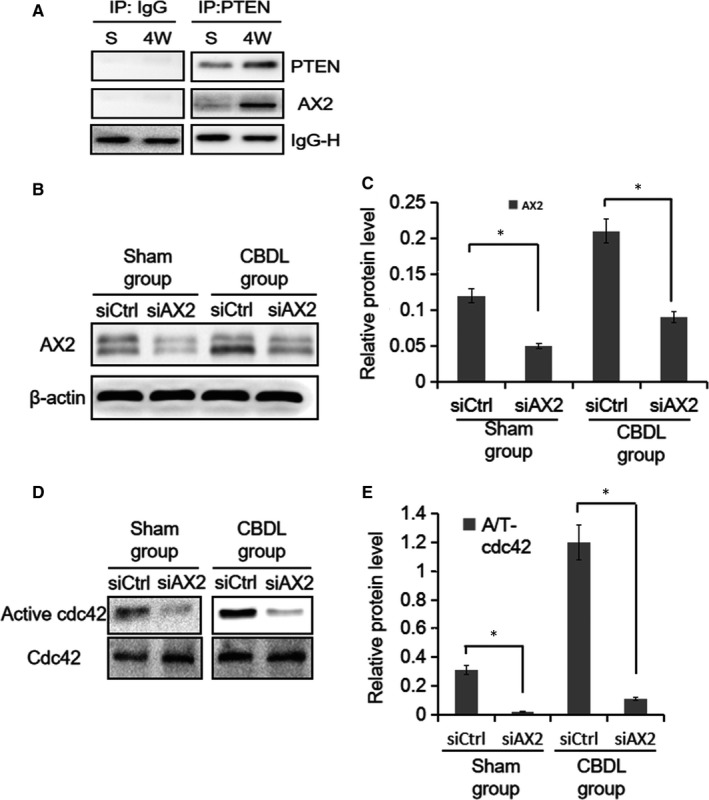
CBDL rat serum‐induced cell polarity defects require AX2. A, Immunoprecipitation assay was performed. It demonstrated that PTEN interacted weakly with AX2 in the sham group, but it showed more robust interaction with AX2 in the CBDL rat serum group. B, Western blot analysis was performed. Following 36 h transfection, AX2 protein expression was strongly suppressed by siAX2. C, The densitometry of AX2 protein expression compared to that of β‐actin is displayed. D, Following 36 h transfection, the activity of Cdc42 was strongly suppressed by siAX2. E, The level of Cdc42 activity normalized to total Cdc42 is displayed. *P < 0.05

It has been shown that PTEN localizes to the apical plasma membrane during epithelial morphogenesis to mediate the enrichment of *PtdIns(4,5)P2* at this domain during cyst development in three dimensional culture. Then, AX2 binds *PtdIns(4,5)P2* and thus is recruited to the apical surface, which in turn recruits Cdc42 to the apical plasma membrane, causing the organization of the sub‐apical actin cytoskeleton and formation of the apical surface and lumen.^26^ Therefore, we checked whether PTEN interacts with AX2 by using Immunoprecipitation assay. PTEN interacted weakly with AX2 in PMVECs stimulated with sham rat serum, but showed robust interaction with AX2 in CBDL group (*P* < 0.05) (Figure 6A). It indicated that the interaction between PTEN and AX2 might be important in the overactivation of Cdc42 in PMVECs stimulated by CDBL rat serum.

Thus, to determine if the PTEN‐mediated increased activity of Cdc42 is dependent on AX2, we partially knocked down AX2 expression in PMVECs via the cell transfection with AX2 siRNA. Western blot analysis revealed that AX2 levels transfected with siRNA‐AX2 were significantly decreased compared to those in cells transfected with scramble sequences (*P* < 0.05) (Figure 6B and C). Interestingly and importantly, decreased levels of Cdc42 activity were detected after 36 hours of transfection with AX2 siRNA in PMVECs stimulated by either CBDL or sham rat serum (Figure 6D and E). Taken together, these results suggested that PTEN activates Cdc42 via its interaction with AX2 in PMVECs.

## 4 DISCUSSION

**Figure 7:**
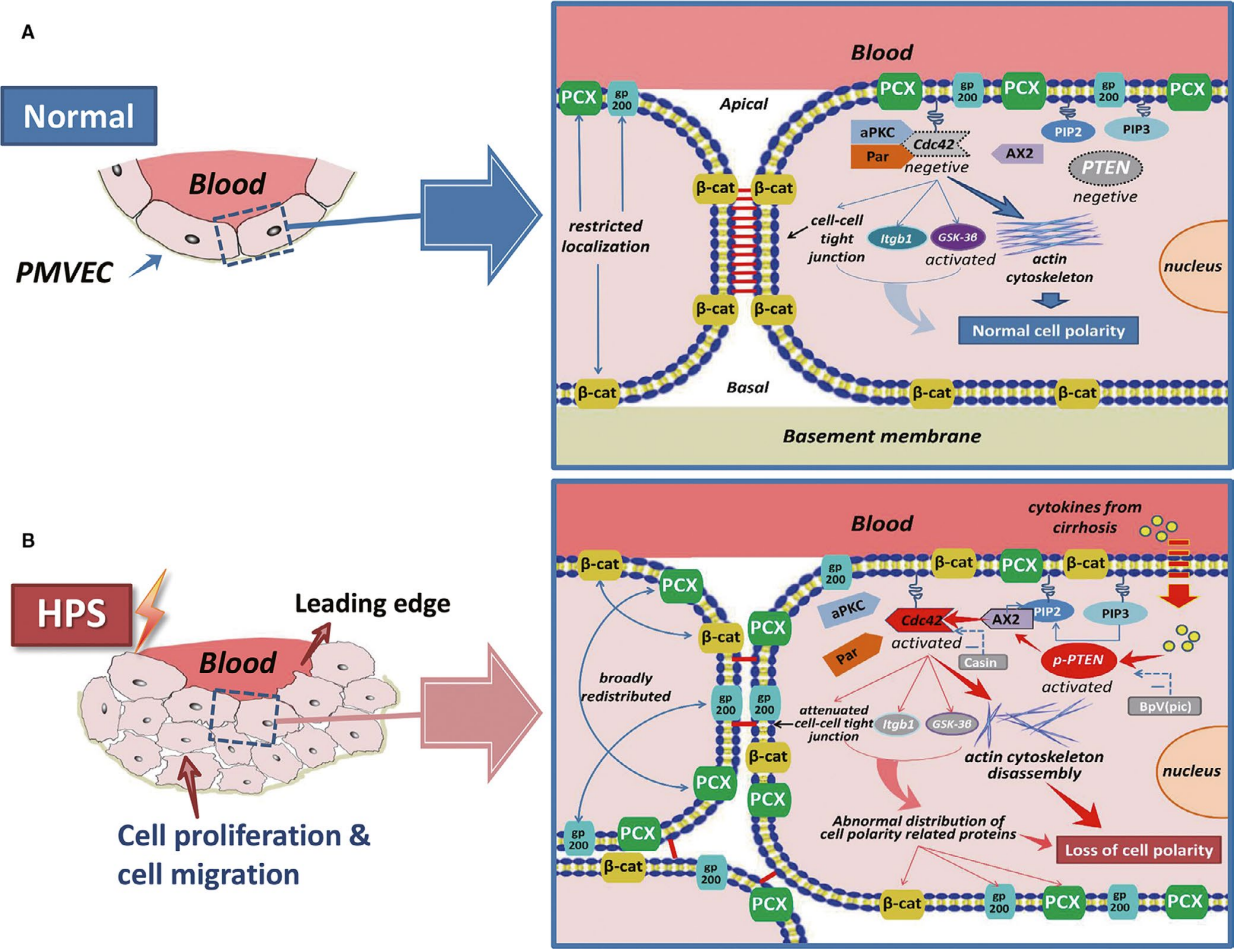
The mechanism of the loss of cell polarity in CBDL rat lung. A, In the pulmonary microvasculature of normal rats, the cells are polarized. Endotheliocyte are arranged in a single layer and are regular in morphology. The cell polarity proteins are regular localized and the cell‐cell tight junctions are strong. PTEN and Cdc42 were in a relatively inactive state. B, In the pulmonary microvasculature of HPS rats, the cells were in a depolarized state. Cells are arranged in multiple layers and are irregularly shaped. The cell polarity proteins are broadly redistributed the cell‐cell tight junctions are weak. Cytokines from HPS rat serum induces the PTEN/AX2/Cdc42 signalling pathway, which promotes actin cytoskeleton disassembly in the lung. Cdc42, as a key upstream regulator, mediates the dysregulation of cell polarity and promotes cell migration and cell proliferation, thereby further participating in the formation process of PVR associated with HPS. Bpv(pic):PTEN inhibitor,Casin:Cdc42 inhibitor

Our previous studies established the role of cell proliferation, migration and phenotype differentiation in PVR associated with HPS.^4^, ^5^, ^6^, ^7^ We noticed that the disruption of apical‐basolateral polarity also leaded to abnormal proliferation and migration of epithelial cells. The disruption of cell apical‐basolateral polarity was classically manifested as dysregulated levels and mis‐localization of normal cell polarity proteins.^27^ In our study, three original localization‐restricted proteins (PCX, β‐catenin and gp200) were examined, and all of them were mis‐located and expanded to all membrane domains in PMVECs treated by CBDL serum in vitro and endothelial cells from CBDL rat lung in vivo, compared with the sham group. We explored the mechanism of the loss of cell polarity in HPS rat lung. Cytotines from cirrhosis induces the PTEN/AX2/Cdc42 signalling pathway, which promoted actin cytoskeleton disassembly in the lung. Cdc42, as a key upstream regulator, mediated the dysregulation of cell polarity and promotes cell migration and cell proliferation, thereby further participating in the formation process of PVR associated with HPS (Figure 7).

Polarity changes the cell‐cycle length, which is known to be associated with cell‐fate changes.^28^ Consistently, we also found that the disruption of endothelial cell apical‐basolateral polarity by CBDL rat serum significantly enhanced the abilities of cell proliferation and migration, together with dramatic rearrangement of the actin cytoskeleton.

It is speculated that the control of cell polarization requires a key molecule that integrates the variety of extra‐ and intra‐cellular signals that are received by the cell. The rearrangement of the actin cytoskeleton also plays a significant role between cell polarity and other cellular activities.^29^

Many previous studies supported the critical role of Cdc42 in control of apical‐basolateral polarity in different systems. The expression of activated Cdc42 mutants can cause filopodia extension as well as alterations in cell shape, migration and morphogenesis. M.Carolina Florian has reported that elevated activity of Cdc42 in aged HSCs is correlates with a loss of polarity. In epithelial cells, the expression of a dominant‐negative Cdc42 mutant (Cdc42T17N) elicited a highly selective loss of basolateral polarity.^30^, ^31^ Furthermore, Cdc42/Par complex activates GSK‐3β and results in destabilization of cell polarity in many diseases.^32^

Previous literatures had reported that PTEN played important regulatory roles in cell migration and proliferation.^33^ It was also worth noting that PTEN played an important role in cell polarity in various diseases.^16^, ^34^ As a dual protein‐lipid phosphatase, PTEN is the major down‐regulator of the pro‐oncogenic PI3K/Akt pathway by degrading PIP3 to an inactive form of PIP2, thus inhibiting Akt activation. In addition, we found that the change of *p‐*PTEN was consistent with that of total PTEN and showed an increase trend in time dependence. This suggested that the increased total PTEN was mainly *p‐*PTEN. Interestingly, it has been shown that AX2 binds *PtdIns(4,5)P2* and is recruited to the apical surface.^26^ Our previous studies had reported that silencing AX2 expression inhibited cell migration and proliferation in the pathogenesis of HPS.^4^, ^5^ Furthermore, PTEN‐mediated segregation of phosphoinositides controls cell polarization through Cdc42, which co‐localizes with the Par/aPKC/Cdc42 complex at the apical plasma membrane of epithelial cells, which is consistent with our results (Figure 6).

Our observations of a continuously active Cdc42 in PMVECs causing the disruption of cell apical‐basolateral polarity provided a novel perspective for the pathogenesis of HPS and also identified the mechanism controlling the regulation of cell migration and proliferation. A deeper and broader understanding of this process will not only identify a new viewpoint for non‐polarized cells with proliferation and metastatic potential but will also identify new strategies to inhibit PVR associated with HPS.

## CONFLICT OF INTEREST

The authors declare no competing interests.
